# Assessing the Quality of an Online Democratic Deliberation on COVID-19 Pandemic Triage Protocols for Access to Critical Care in an Extreme Pandemic Context: Mixed Methods Study

**DOI:** 10.2196/54841

**Published:** 2024-11-11

**Authors:** Claudia Lucrecia Calderon Ramirez, Yanick Farmer, James Downar, Andrea Frolic, Lucie Opatrny, Diane Poirier, Gina Bravo, Audrey L'Espérance, Nathalie Gaucher, Antoine Payot, Joseph Dahine, Peter Tanuseputro, Louis-Martin Rousseau, Vincent Dumez, Annie Descôteaux, Clara Dallaire, Karell Laporte, Marie-Eve Bouthillier

**Affiliations:** 1 Université de Montréal Montréal, QC Canada; 2 Université du Québec à Montréal Montréal, QC Canada; 3 Division of Palliative Care, Department of Medicine, University of Ottawa Ottawa, ON Canada; 4 Hamilton Health Sciences Hamilton, ON Canada; 5 McGill University Health Centre Montréal, QC Canada; 6 CIUSSS du Centre-Sud-de-l‘Île-de-Montréal Montréal, QC Canada; 7 Université de Sherbrooke Sherbrooke, QC Canada; 8 École Nationale d'Administration Publique (ENAP) Montréal, QC Canada; 9 Centre Hospitalier Universitaire Sainte-Justine, Université de Montréal Montréal, QC Canada; 10 Department of Family Medicine and Primary Care, Li Ka Shing Faculty of Medicine The University of Hong Kong Hong Kong SAR China; 11 Polytechnique Montréal Montreal, QC Canada

**Keywords:** quality assessment, online democratic deliberation, COVID-19 triage or prioritization, critical care, clinical ethics

## Abstract

**Background:**

Online democratic deliberation (ODD) may foster public engagement in new health strategies by providing opportunities for knowledge exchange between experts, policy makers, and the public. It can favor decision-making by generating new points of view and solutions to existing problems. Deliberation experts recommend gathering feedback from participants to optimize future implementation. However, this online modality has not been frequently evaluated.

**Objective:**

This study aims to (1) assess the quality of an ODD held in Quebec and Ontario, Canada, on the topic of COVID-19 triage protocols for access to critical care in an extreme pandemic context and (2) determine its transformative aspect according to the perceptions of participants.

**Methods:**

We conducted a simultaneous ODD in Quebec and Ontario on May 28 and June 4, 2022, with a diversified target audience not working in the health care system. We used a thematic analysis for the transcripts of the deliberation and the written comments of the participants related to the quality of the process. Participants responded to a postdeliberation questionnaire to assess the quality of the ODD and identify changes in their perspectives on COVID-19 pandemic triage protocols after the deliberation exercise. Descriptive statistics were used. An index was calculated to determine equality of participation.

**Results:**

The ODD involved 47 diverse participants from the public (n=20, 43% from Quebec and n=27, 57% from Ontario). Five themes emerged: (1) process appreciation, (2) learning experience, (3) reflecting on the common good, (4) technological aspects, and (5) transformative aspects. A total of 46 participants responded to the questionnaire. Participants considered the quality of the ODD satisfactory in terms of process, information shared, reasoning, and videoconferencing. A total of 4 (80%) of 5 participants reported at least 1 change of perspective on some of the criteria and values discussed. Most participants reported that the online modality was accessible and user-friendly. We found low polarization when calculating equal participation. Improvements identified were measures to replace participants when unable to connect and optimization of time during discussions.

**Conclusions:**

Overall, the participants perceived the quality of ODD as satisfactory. Some participants self-reported a change of opinion after deliberation. The online modality may be an acceptable alternative for democratic deliberation but with some organizational adaptations.

## Introduction

### Democratic Deliberation

Democratic deliberation (DD) is a citizen participation and engagement method that is increasingly being applied across various fields of science, including health care. DD has been applied to issues of public interest related to health care, public health, and ethics [1-4]. DD involves the participation of members of the public of a given community for the purpose of collective reflection and discussion regarding a topic of public interest, emphasizing the value of their voice in the reflection. It is an interactive two-way dialogue between nonexperts and experts based on a qualitative methodology [5,6].

DD differs from other public participation methods, such as focus groups and consultations, in three key ways: (1) the provision of information relevant to the policy in question to broaden its understanding; (2) the facilitation of collective reflection and discussion with the participants in an atmosphere that fosters respect, equity, and a common good perspective; and (3) the potential use of participants’ informed views to improve their health policies, especially when dealing with complex and controversial issues, such as a crisis situation [6-10]. Experts in deliberative processes recommend assessing the quality of the DD to obtain participants’ feedback as a means to optimize its future application [11,12].

However, the definition of quality carries a broad spectrum of expectations. In fact, there is no consensus regarding the criteria that a quality assessment must evaluate. Some studies have combined several criteria for face-to-face DD, such as the process, acceptability, reasoning, independence, transparency, reliability, satisfaction, comprehensibility of the task, accessibility, viewpoint transformation, cost-effectiveness, and logistics [11,13-18]. Experts have underlined that there is no ideal standard according to which the quality of a face-to-face DD must be evaluated [11]. DDs have generally been conducted in person; however, during the COVID-19 pandemic, due to public health measures aimed at curbing the spread of SARS-CoV-2, some DDs were conducted online [19].

### Online Democratic Deliberation

Since the 1990s, there has been an increased interest in using online DD (ODD), particularly in social science and in public policy making [20-23]. Interest in using ODD increased during the COVID-19 pandemic [24]. Despite this fact, very few quality assessments of ODD have been published. However, certain dimensions have been evaluated, such as participants’ behavior throughout the process, the content of the discussions, the design (eg, synchronous or asynchronous modality and the role of online facilitators), and participants’ learning and their opinion changes after deliberation [25-37]. We did not find a standardized and validated tool to evaluate the quality of online deliberation, and there seems to be no consensus on the criteria to be considered [38,39].

Although the online modality already existed before the COVID-19 pandemic, it was generally used in a mixed format [40,41]. Few studies made quality comparisons between face-to-face DD and ODD [42-47]. Such comparisons made it possible to identify some of the disadvantages and advantages associated with ODD. For instance, some disadvantages pertained to decreased participant interaction at the beginning of the discussions where more silence was noted compared to face-to-face sessions. Facilitators had to put in extra effort to engage participants in the discussion, as not all participants could be visible on the screen at the same time [42]. This modality also made it more difficult to observe and interpret participants’ nonverbal communication. Translation into other languages has also been complexified with the online modality. Furthermore, technical difficulties and accessibility have been reported as issues with ODD. For instance, some studies highlighted the fact that older people lacked technical skills and lacked internet access in their care homes, which prevented them from participating in an online format [43-45]. Two studies also reported that technical glitches impacted the effectiveness of the process; however, both studies concluded that the 2 modalities ultimately produced similar results [42,44].

Conversely, some advantages have also been highlighted. For example, it has been reported that ODD allows for more time to reflect during discussions and increases participants’ comfort levels. Participants tend to feel less intimidated by others, making them more comfortable in expressing their opinions freely [19]. ODD also promotes diversity and inclusion by eliminating the need for participants to travel, making it accessible to individuals with physical or financial limitations. It also accommodates older adults, who can participate from home with the assistance of caregivers [45,47]. Finally, ODD offers logistical advantages, such as greater scheduling flexibility and the ability to record sessions, which can be reviewed later if needed [46,47].

Given that little is known about the quality of ODD, we aimed to contribute to this knowledge gap by presenting the results of an empirical quality assessment of an ODD regarding triage protocols for accessing critical care in Quebec and Ontario. We prepared our assessment tool according to a validated framework for face-to-face deliberations [11]. We explored the participants’ self-perceived change of opinion regarding the criteria and values that should underpin triage protocols to appreciate the transformative dimension of DD [48]. This study is one of the few ODDs conducted to obtain the public’s perspectives on triage protocols in extreme pandemic contexts while involving participants in the evaluation of this type of methodology.

## Methods

### Design

This study used a convergent mixed methods design. Thus, it involved collecting both qualitative and quantitative data simultaneously, analyzing the 2 sets of data separately, and then merging the results of the 2 sets of data analyses for comparison purposes. The data then complemented each other, enriching our understanding of citizens’ perspectives on the quality of the deliberative exercise [49]. The topic discussed during the ODD consisted of COVID-19 triage criteria for access to critical care in extreme pandemic contexts. However, the focus of this study pertains to the quality evaluation of the ODD. The findings of this quality assessment have not been reported elsewhere.

### Target Population and Recruitment

The target population was the members of the public from the provinces of Ontario and Quebec.

For the recruitment of participants, we had the collaboration of the Institut du Nouveau Monde (INM), an independent, nonpartisan organization dedicated to increasing citizen participation in democratic life. INM worked in coordination with Leger Opinion, an online polling firm, which conducted participant screening in both provinces. Participants were initially reached via a call for applications posted on Leger Opinion’s website, targeting a pool of 250 candidates from each province. Leger Opinion collected the application forms from April 4 to April 24, 2022. Leger Opinion subsequently conducted a random preselection of applications submitted by the public registered on its website, which resulted in 197 applications from Ontario and 202 applications from Quebec. A second semirandom selection of candidates was carried out by the INM to ensure the diversity of both groups of participants with respect to their demographic variables and region of origin. The first stage of this second selection process consisted of evaluating each application according to the inclusion criteria. The second stage consisted of selecting the 60 final candidates (n=30, 50% from each province) randomly until the composition of each group met the diversity criteria. The inclusion criteria were as follows: citizens of Ontario and Quebec aged >18 years, fluent in either English or French, and possessing basic online participation skills with access to high-speed internet. People studying or working in the field of health care and social services were excluded from this deliberation. The goal was to obtain an outside perspective from a community unrelated to the health care system.

Application forms compiled by Leger Opinion were also designed to collect demographic data on the prospective candidates. INM used these data to carry out the proportional and final selection. Furthermore, these data were used to document the characteristics of the participants in this study. The research team prepared a consent form in both English and French. INM sent and collected this consent form as well as confirmed the participation of each of the candidates on May 2, 2022. Some people were contacted to be placed on a list of substitutes to participate in the event of a withdrawal.

### ODD Procedure

**Figure 1:**
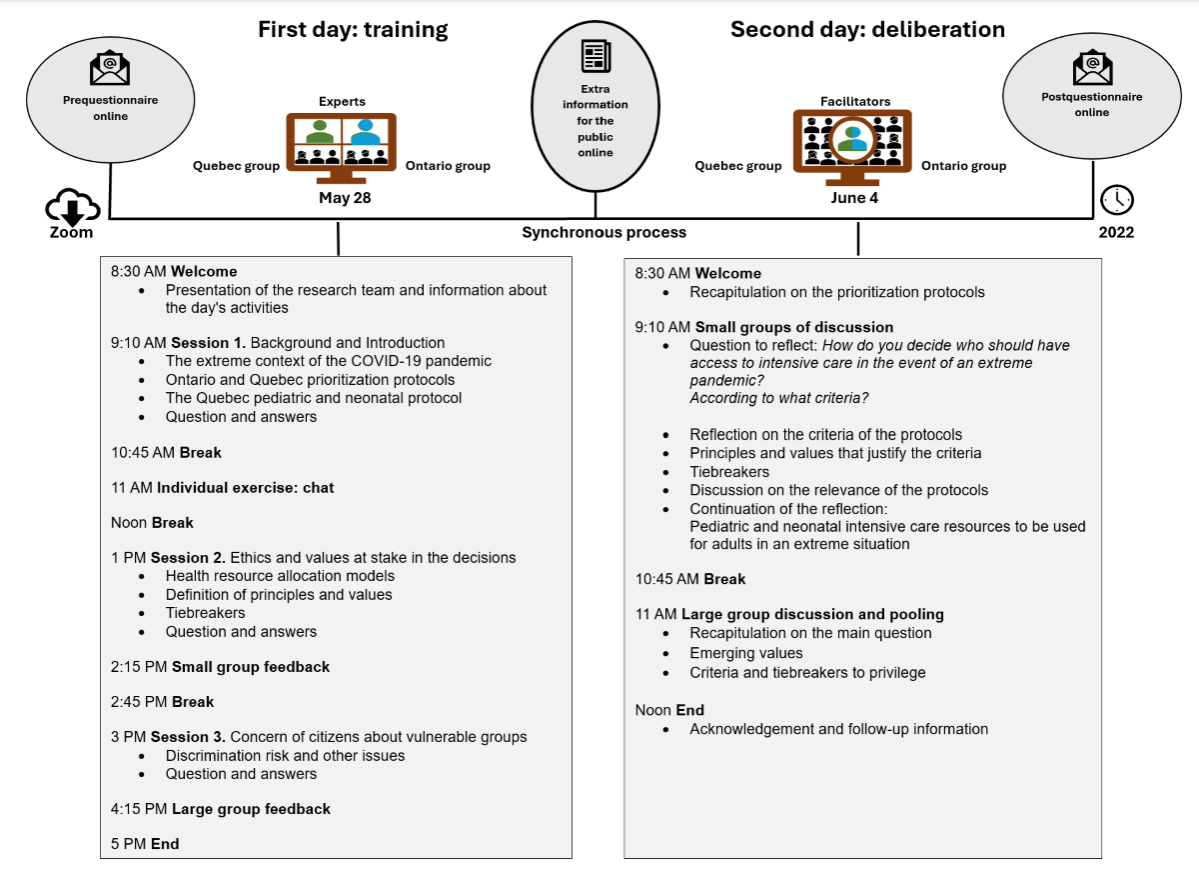
Overview of the online democratic deliberation process.

An overview of the ODD process is presented in Figure 1. In summary, the ODD took place for 2 days on the Zoom (Zoom Video Communications) platform, simultaneously in Quebec and Ontario (Canada), on May 28 (training session) and June 4 (deliberation session), 2022. Participants received an online information document on the main concepts to facilitate understanding and encourage their participation before the deliberation.

Members of the research team and the INM carefully designed the program of presentations for the training session to equip participants for the discussion during the deliberation. Experts from various disciplines oversaw the training session. This session consisted mainly of topics related to the COVID-19 pandemic triage, its context, and its ethical issues. Experts who collaborated in the presentations were 2 intensive care physicians, 2 pediatricians, 2 ethicists, and 2 university professors in community participation and patient partnership. To ensure that all participants received the same training, the experts’ presentations were identical in both English and French. The experts were instructed to present their content and then respond to questions until all inquiries had been addressed. INM facilitators oversaw the deliberation session, which included small group discussions and a plenary discussion where participants reflected and voiced out their perceptions regarding triage protocols for access to intensive care in the COVID-19 pandemic, their criteria, and ethical values to prevail. Consensus building was intended but not required. More details regarding recruitment and the overall ODD process have been reported previously [50].

### Data Collection

Data collection took place in 2 ways: during the deliberation process and through a postdeliberation online questionnaire. Members of the research team stored all the data collected from this study in the University of Montreal’s OneDrive (Microsoft Corp) system to ensure confidentiality.

#### During the Deliberation Process

We collected the participants’ perceptions about the quality of the process at the end of both the training and deliberation sessions. Members of the research team and the INM recorded the entire process of the ODD on the Zoom platform. These recordings were then transcribed in both languages. A member of the team listened to the recordings on 2 occasions to compare and check the accuracy of the verbatim transcripts. These verbatim transcripts were deidentified before conducting the analysis. Observation notes were also taken throughout the deliberation session on preformatted templates; however, these notes only captured information about the topic of the deliberation (triage) and not about the quality of the process.

#### Postdeliberation Questionnaire to Assess the Quality of the ODD

We modified the assessment framework proposed by De Vries et al [11] to evaluate 3 important dimensions: the process, the information, and the reasoning from the point of view of the participating public. Considering the online mode of the deliberation, it was necessary to make some modifications to this evaluation in three ways: (1) by adding questions addressing the visual, sound, and usability aspects of the online modality; (2) by adding an open-ended question at the end for quality feedback; and (3) by restructuring some questions to obtain more comprehensive information from participants during the online interface, trying to keep the evaluative objective of the original framework.

We pretested the questionnaire with the help of 7 volunteers to evaluate whether the questions were intelligible, unambiguous, and unbiased. Once the pretest was undertaken, the questionnaire was re-evaluated and optimized by coresearchers with experience in qualitative and quantitative research. The questionnaire was redrafted according to the feedback provided, and then, we integrated it into the LimeSurvey software (Carsten Schmitz and LimeSurvey team) provided by the University of Montreal.

This questionnaire contained questions to assess the quality and questions related to the participants’ postdeliberation perception change. The questions were primarily close-ended questions with Likert-type scales and an open-ended question at the end to allow space for feedback. Details are available in Multimedia Appendix 1.

Participants were emailed the link to the questionnaire on June 17, 2022, and were given a period of 2 weeks to complete it. Two members of the research team collected and deidentified the data from the questionnaire. Data obtained from the closed-ended questions were separated from those obtained from the open-ended questions and archived in their respective log files for management.

#### Tool to Assess Equality of Spoken Interventions

We applied the Herfindahl-Hirschman Index (HHI) to determine the contributions of each participant during the deliberation session. Other deliberation studies have used this index to estimate the equality of interventions among participants and to detect the presence of polarization in the group’s dynamics. This index was originally used in marketing and is now also used in disciplines related to ethics [51,52].

We used the transcripts collected to calculate the HHI in both groups, determine whether some participants dominated in terms of their interventions, and detect little or no participation. One member of the team was responsible for collecting the transcripts from the participants, counting them, and entering them into the online calculator. We calculated this index using the online HHI calculator [53]. Details of the calculations are available in Multimedia Appendix 2.

### Data Analysis

#### Qualitative Analysis

We carried out a thematic analysis using the transcriptions of the ODD sessions and the written comments (open-ended questions) from the questionnaire related to the quality. We integrated the transcripts and the participants’ written comments into the NVivo software (version 14; Lumivero), released in 2023. Two independent coders reread the qualitative data to become familiar with the content. Through inductive coding, they identified the main emerging themes focusing on the quality of the deliberation process. One of the coders created a codebook to organize codes into themes and subthemes. Members of the research team reviewed the codebook on 2 occasions to ensure that the coding was a reliable representation of the data obtained regarding the quality of the ODD. They discussed coding differences until a consensus was reached. Two members of the research team calculated the percentage of agreement between the coders using NVivo, which generated a κ score of 0.8.

#### Quantitative Analysis

For this analysis, we included only the answers to the closed-ended questions that pertained to the quality of ODD and the self-perception of opinion changes. For the quantitative statistical analysis, we used SPSS software (version 21.0; IBM Corp). Our analysis was only descriptive, which included frequencies, percentages, means, and SDs. We calculated the response rate to the survey as a percentage. For the analysis of the quality assessment questionnaire, we adhered to the evaluative objectives of the framework proposed by De Vries et al [11]. The quality assessment survey included 15 items answered on a 5-point Likert scale (eg, 1=very easy and 5=very difficult). We analyzed the frequencies and percentages of each of the items related to process, information, reasoning, and videoconferencing. Participants’ responses were rated as positive on Likert scales 1 and 2, neutral on Likert scale 3, and negative on Likert scales 4 and 5. For interpreting the mean scores, a score close to 1 indicates a positive evaluation of each aspect, while a score close to 5 indicates a negative evaluation of the assessed variables. Similarly, we analyzed frequencies and percentages of each of the items in the question on participants’ self-assessment of perspective changes. This question included 4 items related to the criteria, principles, and values contained in the adult and pediatric triage protocols. These items were rated using a 4-point Likert scale score (1=totally and 4=not at all). Participants’ responses were rated as having experienced a lot of, some, or little change of perspective on Likert scales 1, 2, and 3, respectively, and were rated on Likert scale 4 as having experienced no change of perspective after the deliberation exercise. For these responses, we interpreted the mean as follows: a score close to 1 indicated a positive evaluation of each of the aspects, while a score close to 4 indicated a negative evaluation of the assessed variables.

For the HHI analysis, we applied the normalized HHI^N^ formula to the online calculations for each group of Quebec and Ontario participants. This formula estimated the presence or absence of polarization during the deliberative session and allowed us to compare the 2 different size groups.

Our interpretation of the formula results was that normalized HHI^N^=0 indicated complete equality of the spoken intervention, and HHI^N^=1 indicated complete polarization of the deliberation dialogue. Details on using the normalized HHI^N^ formula for assessing the equality of interventions are provided in Multimedia Appendix 2.

### Ethical Considerations

This study was approved by the Comité d’éthique de la recherche en sciences et en santé, de l’Université de Montréal on March 15, 2022 (no. 2022-1466), and by the Bureau d’éthique et d’intégrité de la recherche de l’Université d’Ottawa on March 28, 2022 (project H-03-22-8010). All participants provided informed consent before participating. Compensation of CAD $200 (US $130) was offered to each participant for their full participation in the process.

## Results

### Quality of the ODD: Thematic Analysis

A total of 47 participants (n=27, 57% from Ontario and n=20, 43% from Quebec), with a diverse demographic representation, took part in the quality assessment at the end of each session. When comparing the places of origin, the highest participation was from la Capitale Nationale, with 4 (20%) out of 20 participants, and Greater Toronto, with 12 (44%) out of 27 participants. Regarding the population groups, of the 47 participants, 2 (4%) were African American, 1 (2%) was Arab, 3 (6%) were Asian, 2 (4%) were Latin American, 2 (4%) were South Asian, and 2 (4%) belonged to First Nations. One (5%) of the 20 participants from Quebec and 2 (7%) of the 27 participants from Ontario reported belonging to a visible group and visible or multiple minority groups, respectively. In terms of work occupancy, 13 (65%) out of 20 participants from Quebec were employed, and 11 (41%) out of 27 participants from Ontario were employed. Regarding participants’ educational level, the most frequent group in Quebec was professionals with preuniversity training (general and technical), with 8 (40%) out of 20 participants, and that in Ontario was participants with a bachelor’s university education, with 9 (33%) out of 27 participants. Regarding annual income, in Quebec, the most frequent income bracket (CAD $ = US $1.30) was between CAD $39,999 and CAD $49,999, and the least frequent income bracket was >CAD $100,000. In Ontario, the most frequent income bracket was between CAD $49,999 and CAD $59,999, and the least frequent income bracket was <CAD $30,000. More details of the demographic characteristics of the participants are described in a previous publication [50].

Five themes emerged from our analysis: (1) process appreciation, (2) learning experience, (3) reflecting on the common good, (4) technological aspects, and (5) transformative aspects. Some quotes from participants have been stated in the subsequent sections for each of the 5 themes.

### Process Appreciation

#### Overview

This theme emerged when coding all the transcripts and the written comments pertaining to participants’ lived experiences during the exercise; for instance, feelings of satisfaction in sharing their perceptions, being listened to, being respected, and even their views on the presentations and the deliberation exercise. We classified these experiences either as positive or negative. Quotations regarding the appreciation of the ODD obtained during the deliberation processes and written comments on the questionnaire were all found positive. Two positive aspects stood out: an excellent experience and sharing with a diverse audience in a respectful atmosphere that encouraged freedom of expression.

#### Positive Quotes

Participants in both groups stated that they had an excellent experience during both the training session and the deliberation session:

I found the presentations excellent.

Participant in Ontario 21

Very helpful. The presenters were great. The presentations were excellent but also difficult. There was so much information that was an important part of our life.

Participant in Ontario 3

I just want to say that I thought the presentations were all wonderful and that I learned a lot. Everything was good. I loved it. I can’t wait for next Saturday.

Participant in Ontario 13

Everything was great even with the long session it did not feel like it was 8 hours.

Participant in Ontario 12

I really enjoyed this experience, the group was great, it was rewarding, a big thank you.

Participant in Quebec 13; translated from French

...But overall, I have to say that the experience was rewarding...

Participant in Quebec 17; translated from French

Participants felt comfortable, respected, and free to express their opinions with a diverse audience:

I really enjoyed being a part of this and offering my opinions and accepting others and no arguments happened and everyone was engaged.

Participant in Ontario 13

As one of the participants remarked at the end of the first session, it was pleasantly surprising to see people from different backgrounds, with different points of view, discussing in mutual respect.

Participant in Quebec 17; translated from French

Excellent choice of having people from different age groups and different parts of province with the opinions and possible obstacles.

Participant in Ontario 16

Very good quality of conferences and debates. Good conviviality and good animation...

Participant in Quebec 14; translated from French

And one thing that I appreciated as well was the smaller groups, because it gives more time for each individual to give their point of view. And then once we get back to the bigger group, we can give a consensus. So, everyone can get their point across within a shorter amount of time than if we stayed in a big group and discuss everything.

Participant in Ontario 8

### Learning Experience

#### Overview

This theme referred to participants’ perceptions related to the overall learning experience during the exercise and the influence of this learning on their own principles and values. More specifically, this theme included comments on the acquisition of knowledge regarding triage during the COVID-19 pandemic, its implications, and the protocols presented. Participants stated that they had learned from the information session and better understood the underlying criteria and values contained in the triage protocols:

Before we even started the meeting, I appreciated that a questionnaire was sent to us because it got us into the mindset of what type of questions we would be asked. And also, especially the first presentation, I think it should be repackaged and broadcast to the masses to explain why we need triage. And once people understand that, then maybe more people will understand why COVID was such a serious situation and why we insisted on vaccines, masks and all that stuff...

Participant in Ontario 8

Totally enjoyed participating. Very well organized, speakers were amazing, and I certainly learned a lot about the behind the scenes during a pandemic.

Participant in Ontario 22

I felt privileged to have access to this information, and to know the scientific and other issues.

Participant in Quebec 3; translated from French

It allowed me to question some of my beliefs and values, I loved it.

Participant in Quebec 13; translated from French

### Reflecting on the Common Good

#### Overview

This theme included participants’ perceptions of their ability to reflect collectively rather than individually on the points raised during the deliberation exercise. Some participants commented that, by sharing their opinions related to the triage protocols and thinking collectively, they were engaged in a reflection for the good of the community; however, 1 participant highlighted that this was not always the case:

...I was surprised to see how many people bring the debate back to their personal situation, despite the explanations on the collective which must be a priority. I perceived a radical change of perception for some, following the explanations, who seemed to understand the priority of the collective, and [I was] disappointed to see that some maintained personal positions...

Participant in Quebec 3; translated from French

Great sense of community with the group.

Participant in Ontario 3

I really enjoyed being a part of this and offering my opinions and accepting others and no arguments happened and everyone was engaged.

Participant in Ontario 13

### Technological Aspects

#### Overview

This theme emerged from the participants’ perceptions of the ease or difficulty of participating in an online deliberation, specifically concerning the technology used. More specifically, participants were asked to comment on aspects such as the use of the Zoom platform, the use of breakout rooms for small group discussions, the plenary sessions, the videoconference, the aspects of volume, visibility, and the internet signal. This theme was subdivided into positive and negative quotes.

#### Positive Quotes

Tech aspects were handled very well. Seamless transition from plenary to breakout groups and back. Top quality presentations and content.

Participant in Ontario 20

I learned lots about Zooming. Unfortunately, my old machine does not support Zoom, so I had to use a pad and it was a learning experience for me, but I did manage.

Participant in Ontario 19

I have never been involved in video conferencing before where I needed to speak so finding out my mic didn't work was very frustrating. The team tried to help as best they could from a distance and I'm very grateful for their attempts. I made the best of it and included any of my opinions in the chat to be recorded or thumbs up on screen if my response was needed quickly.

Participant in Ontario 3

This is a VERY well-run study, apart from some people had technical difficulties (inevitable), I'd give 4.8 out of 5 for the whole process. Presenters are all experts in their field, presentation very informative, group discussions are well organized and purposely remixed which is beneficial.

Participant in Ontario 7

Nothing can replace face to face meetings; however, they did the best they could with this format.

Participant in Ontario 21

#### Negative Quotes

On the links that were given on the chat, I was not able to open it on my iPad, I had to copy and paste in browser.

Participant in Quebec 20; translated from French

Unfortunately, the sound quality from some of the presentations was lacking. I think this was due to some technical issues, but the team adapted and carried on with the seminars as best they could. At the end of the day, I was able to understand all of the information presented.

Participant in Ontario 17

### Transformative Aspects

#### Overview

This theme emerged when coding the changes perceived by participants on the topics discussed, such as triage protocols in the COVID-19 pandemic and the criteria and values, considering the transformative nature of the deliberations. Participants commented on the changes they experienced with respect to their initial perspectives after the deliberation process. These expressions were divided into positive change, negative change, and no change.

#### Positive Change

I did not understand how complex this was, I feel I learned a LOT more and it helped me also respect their positions a lot more.

Participant in Ontario 12

I used to completely disagree with this type of protocol but now I see it as a requirement.

Participant in Quebec 16; translated from French

I didn’t think the discussion was this advanced. I changed my perspective when I learned that non-ICU patients were not left to their own devices.

Participant in Quebec 9; translated from French

#### Negative Change

I thought a protocol was a good thing, but after looking at it and discussing it I feel that it is very much for the benefit of the healthcare workers. I don’t feel like it serves any other purpose and is extremely cumbersome on the administrative side.

Participant in Quebec 5; translated from French

#### No Change

...I did not change my thinking for the tiebreaker situation, in fact I'm more convinced that lottery is much better way to handle tiebreakers...

Participant in Ontario 7

### Written Comments on the Questionnaire

Written comments (open-ended questions) on quality were provided by 12 (60%) out of 20 participants from Quebec and 12 (44%) out of 27 participants from Ontario. The trend of responses on quality is presented in Multimedia Appendix 3. Written comments on the change of perspective were provided by 11 (55%) out of 20 participants from Quebec and 6 (22%) out of 27 participants from Ontario. The trend of responses in terms of participants’ self-perceived change of opinion is presented in Multimedia Appendix 4.

### Quality of the ODD: Questionnaire

Out of 47 participants, 46 answered the questionnaire (the level of participation was 98%). Most participants responded positively to each of the evaluated aspects, which included an evaluation of the process itself, the information shared with the participants, the collective reasoning experience, and the videoconference (Table 1).

Two aspects of the process stood out: the perception of equal opportunity to share opinions and feeling respected during the deliberation, both presenting 45 (98%) positive responses out of 46 responses. Positive responses were also found in other dimensions, such as commitment during the discussion, with 44 (96%) positive responses out of 46 responses, and facilitation, with 39 (85%) positive responses out of 46 responses.

Furthermore, we found positive responses with respect to the information shared with participants during the information session. A positive response rate of 100% (46/46) was obtained for both the learning experience and comprehension dimensions, indicating total satisfaction for both. In terms of the impact of the information on participants’ opinions, the positive responses were 41 (89%) out of 46 responses. The use of correct information by participants was found to have 38 (83%) positive responses out of 46 responses, which suggests that they received and shared more correct than incorrect information. The lowest percentage found among the positive responses pertained to the consultation of experts, with only 20 (43%) positive responses out of 46 responses, which indicates that participants did not frequently consult the experts.

Regarding the evaluation of collective reasoning, the responses were generally positive. The reflection for a common good or societal perspective showed 46 (100%) positive responses out of 46 responses. A total of 31 (94%) positive responses out of 33 responses were reported for the justification of their opinions, and 43 (93%) positive responses out of 46 responses were reported for participants’ openness to the complex and difficult issues discussed in the deliberation.

Regarding the evaluation of the videoconference, 46 (100%) positive responses out of 46 responses were found for the sound quality, 45 (98%) positive responses out of 46 responses for the video quality, and 42 (95%) positive responses out of 44 responses for the ease of using the online videoconference for deliberation.

**Table 1:** Assessing the quality of an online democratic deliberation on the COVID-19 pandemic triage (N=46).

Evaluated aspects and questions			Respondents, n (%)		Criteria, n (%)						Scores, mean (SD)
**Process^a,b^**											
	Facilitation: how did you perceive your participation in the deliberation or sharing your perspectives in a videoconference?	46 (100)		Very easy, 24 (52)		Easy, 15 (33)	Neutral, 2 (4)	Difficult, 5 (11)	Very difficult, 0 (0)	1.74 (0.976)	
	Equal participation: how did you perceive the opportunity to share your opinions and ask questions during the process?	46 (100)		Very equal, 33 (72)		Equal, 12 (26)	Neutral, 0 (0)	Unequal, 1 (2)	Very unequal, 0 (0)	1.33 (0.598)	
	Respect: did you feel respected in sharing your opinions with other participants in the process?	46 (100)		Very respected, 32 (70)		Respected, 13 (28)	Neutral, 1 (2)	Little respected, 0 (0)	Not respected, 0 (0)	1.33 (0.51)	
	Commitment: how would you rate your engagement with the discussion group in the process?	46 (100)		Very committed, 32 (70)		Committed, 12 (26)	Neutral, 2 (4)	Little committed, 0 (0)	Uncommitted, 0 (0)	1.35 (0.56)	
**Information^a,b^**											
	Expert consultation: how often do you estimate that you have consulted experts during the deliberation to obtain clarification?	46 (100)		Very frequent, 3 (7)		Frequent, 17 (37)	Neutral, 12 (26)	Infrequent, 14 (30)	Not frequent, 0 (0)	2.80 (0.95)	
	Use of incorrect information: do you consider that you received or shared incorrect information during the deliberation?	46 (100)		Not at all, 27 (59)		Not really, 11 (24)	Neutral, 3 (6)	A little, 3 (7)	Totally, 2 (4)	1.74 (1.12)	
	Learning new information: how do you perceive your learning from the information obtained in the deliberation?	46 (100)		Very satisfied, 37 (80)		Satisfied, 9 (20)	Neutral, 0 (0)	Dissatisfied, 0 (0)	Very dissatisfied, 0 (0)	1.20 (0.40)	
	Understanding and applying the information: how would you rate your understanding of the information presented in the deliberation?	46 (100)		Very clear, 32 (70)		Clear, 14 (30)	Neutral, 0 (0)	Unclear, 0 (0)	Very unclear, 0 (0)	1.30 (0.46)	
	Impact of information on opinions: how do you perceive the impact of deliberation information on your opinions?	46 (100)		Very influential, 13 (28)		Influential, 28 (61)	Neutral, 4 (9)	Little influential, 1 (2)	Not influential, 0 (0)	1.85 (0.66)	
**Reasoning^a,b^**											
	Rationale for opinion: how would you rate your shared opinions in the deliberation? Were your opinions justified?	33 (71)		Very justified, 0 (0)		Justified, 31 (67)	Neutral, 2 (4)	Poorly justified, 0 (0)	Not justified, 0 (0)	2.06 (0.24)	
	Openness to complexity: how would you rate your openness to the difficult topics discussed in the deliberation?	46 (100)		Very open, 27 (59)		Open, 16 (35)	Neutral, 3 (6)	Little open, 0 (0)	Not open, 0 (0)	1.48 (0.62)	
	Consideration of societal perspective: how did you perceive your consideration of the collective perspective or thinking for a common good during the deliberation?	46 (100)		Very considered, 26 (57)		Considered, 20 (43)	Neutral, 0 (0)	Poorly considered, 0 (0)	Not considered, 0 (0)	1.43 (0.50)	
**Videoconference^c^**											
	How would you rate the audio quality or sound quality?	46 (100)		Very satisfied, 35 (76)		Satisfied, 11 (24)	Neutral, 0 (0)	Dissatisfied, 0 (0)	Very dissatisfied, 0 (0)	1.24 (0.43)	
	How do you rate the video quality or image quality?	46 (100)		Very satisfied, 38 (83)		Satisfied, 7 (15)	Neutral, 1 (2)	Dissatisfied, 0 (0)	Very dissatisfied, 0 (0)	1.20 (0.45)	
	What is your assessment of the ease of use of videoconferencing for deliberation?	44 (95)		Very easy, 34 (74)		Easy, 8 (17)	Neutral, 2 (4)	Difficult, 0 (0)	Very difficult, 0 (0)	1.27 (0.54)	

^a^Questions developed according to the framework proposed by De Vries et al [11].

^b^Modified questions.

^c^Added questions.

### Changes in Perspectives Reported by Participants

Regarding the evaluation of the postdeliberation changes in participants’ perspectives, based on the questionnaire (close-ended questions), we observed a similar change for all the evaluated aspects (Table 2).

In relation to the criteria contained in the triage protocols, 14 (30%) out of 46 participants reported a total change of opinion, 19 (41%) reported a partial change, and 8 (17%) reported at least 1 change. Thus, 41 (89%) out of 46 participants reported some changes in their perspective after deliberation. Results were similar for the other aspects consulted. A total of 39 (85%) out of 46 participants reported some changes of perspective regarding the principles and values of the adult protocol and the tiebreakers, and 40 (87%) out of 46 participants reported a change regarding the principles and values of the pediatric and neonatal protocol. Participants who reported experiencing no change in perspectives after deliberation consisted of the smallest group.

**Table 2:** Identifying the evolution of the public perspectives on the COVID-19 pandemic triage protocols after deliberation (N=46).

Question: do you consider that this democratic deliberation has generated a change in your perspectives? Please check your degree of change in perspective regarding each of the following options.	Totally, n (%)	Partially, n (%)	Somewhat, n (%)	Not at all, n (%)	Scores, mean (SD)
Clinical criteria of the adult protocol	14 (30)	19 (41)	8 (17)	5 (11)	2.09 (0.96)
Principles and values of the adult protocol	11 (24)	18 (39)	10 (22)	7 (15)	2.28 (1.00)
Pediatric and neonatal protocol principles and values	13 (28)	14 (30)	13 (28)	6 (13)	2.26 (1.02)
Tiebreaker criteria	15 (33)	15 (33)	9 (19)	7 (15)	2.17 (1.06)

### Estimation of Equality of Spoken Intervention

In calculating the HHI, we were able to obtain an estimate of the spoken intervention of both groups of participants. Results indicated a low polarization in the group dynamics for both Quebec and Ontario groups (Table 3). We observed that a few participants intervened more and that a few others did not intervene, which was similar in both groups. Details are available in Multimedia Appendix 2.

**Table 3:** Summary of the Herfindahl‐Hirschman Index (HHI) and normalized HHI of the participants during deliberations (N=47).

Measure	Quebec group	Ontario group
Normalized HHI	0.035	0.033
HHI	688	830
Range of spoken intervention (%)	0-15	0-12
Participants, n (%)	20 (43)	27 (57)

## Discussion

### Principal Findings

This study found a positive appreciation regarding the ODD process in both the Quebec and Ontario groups, and minimal differences were observed regarding the equality of participation in both groups based on the HHI scores. Participants in both provinces voiced favorable perspectives in terms of the quality of the ODD. This deliberation exercise demonstrated that participants could learn specific information about triage protocols, some of whom probably had prior misconceptions about them. Despite the sensitivity of this topic, the participants showed openness in trying to understand complex ethical issues. We were aware that it would not be easy for them; however, they demonstrated their ability to grasp the shared information and to reflect on ethical dilemmas associated with the topic at hand. Similar findings were found in a study conducted with the public regarding surrogate consent for research in people with dementia. In this study, the topic was also sensitive in nature, and the participants also demonstrated an openness to the complexity of the issues involved [54].

As for reflecting on the common good, we noticed a certain difficulty at the beginning of the deliberation process for a few participants. However, during the process and especially at the end, we observed a change in the participants’ perspectives. They adopted a vision that was more focused on the common good. In multicultural societies, it is common to find a diversity of values that influence people’s perspectives. This heterogeneity of thoughts provides richness in terms of considering multiple angles and moral values on the subject to treat [55]. One might think that this pluralism would run counter to a common vision, but this was not the case. The breakout sessions enabled participants to delve more deeply into the subject, leading to fruitful exchanges and a sharing of diverse viewpoints. The diversity of the participants in terms of sociodemographic characteristics and the pluralism of the values discussed did not create schisms between them. Instead, participants rallied around common values and worked together to bring out what they considered to be the best possible solutions for triaging access to intensive care in the extreme context of the COVID-19 pandemic. We believe that most participants felt that they were engaged in a collective reflection, which is an important aspect to achieve during these processes [56,57].

Conducting this ODD in a COVID-19 pandemic context was a new challenge for the research team and the participants. Surprisingly, the participants felt comfortable with the online modality. Some technical problems were reported by 2 (4%) out of 47 participants, one related to the loss of sound and the other related to problems in the manipulation of the smart device, but these issues were rapidly resolved. We believe that the health measures in place during the COVID-19 pandemic also facilitated the adaptability of the participants to the new technology. The advanced age of some participants, as well as their low level of education or income, did not prove to be an obstacle to their online participation. Other studies have previously noted that older adults often have a limited ability to handle technology, which can create challenges for their participation [43-45]. An adequate internet signal is of vital importance to carry out ODD. In this study, we were affected by this type of barrier, losing some participants in the first session due to failures in their internet signal, a situation beyond the control of the research team (bad weather). We consider this to be the most problematic aspect of an online modality. The ODD was conducted in French for the Quebec group and in English for the Ontario group to provide the same content in both languages and to facilitate the participants’ understanding, thus avoiding problems of linguistic interpretation [42].

Some studies mentioned “Zoom fatigue” during videoconferences [42,58]. Previous efforts to prepare participants to effectively use the Zoom platform and the screen were useful, for example, keeping their cameras on, minimizing screen sharing, using emoticons to express themselves, and asking for their turn to talk. Some participants expressed their appreciation related to the small group discussions, the chat between discussions, and the breaks during the process. We believe that the facilitators’ animation made the discussions more enjoyable and reduced “Zoom fatigue.” Some argue that it is more difficult to observe the faces, gestures, as well as verbal and nonverbal cues during an online meeting, which are easier to observe in person [42]. We were not able to quantify this aspect, but the facilitators did take it into account, especially during the small group discussions. The facilitators initially asked questions in a general way and, in some cases, in a direct way to each participant to encourage their participation and to prevent a passive attitude or, on the contrary, a polarization in the participation. Therefore, the role of the facilitators was essential [42,44]. Regarding the equality of spoken intervention according to the estimated HHI, both groups obtained a similar score, showing a low tendency of polarization during the group discussions on the day of the deliberation. This suggests that the facilitators tried, as much as possible, to achieve an equal distribution of participation [59].

Some participants reported having experienced at least 1 change of perspective in relation to the protocols presented. However, our objective was not to direct efforts to change the participants’ perspectives on the triage protocols. We were simply interested in finding out whether these perspectives had been modulated or affected by the deliberation process. The public may be susceptible to misinformation due to a lack of complete and accurate information [60,61]. DD has proved to be an opportunity to complement and provide reliable information to the public on health strategies during the COVID-19 pandemic [62]. A deliberation conducted both in person and online found that participants’ opinions and behaviors had changed. Some participants had increased their knowledge and felt more committed and encouraged to participate in deliberation online processes [29]. Others not only changed their opinions but also strengthened their sense of civic engagement [63]. In an ODD conducted in Finland about new transportation systems for the community and urban planning, the results showed that participants experienced a remarkable change of perspectives at the end of the process [64]. These results suggest that ODD can be transformational for the participants.

Some key organizational aspects are essential to achieve an effective contribution from participants, such as encouraging participation from all deliberators, adhering to the schedule, and ensuring a stable internet connection. Having backup participants in case some experience problems with their internet connection could be helpful to avoid losing participants. However, in our study, it was not possible to contact other participants to replace those experiencing technical problems, as this problem was not immediately reported. Nevertheless, the team was able to replace 1 participant in the initial hours of the training day due to an emergency. Finally, it is important to properly save the Zoom records of all the sessions (plenary and breakout sessions) because, in this study, 1 member of the team faced technical difficulty in recording 1 of the sessions on the Zoom platform.

### Limitations

Regarding the limitations of this study, our participants did not constitute a representative and proportional quantitative sample of the populations studied. Therefore, our results cannot be generalized, and this should be considered when interpreting our results. Furthermore, we recognize that an online modality limited us to recruiting only people with internet access and minimal basic computer skills. However, we believe that it allowed us to foster inclusiveness of participation and citizen engagement in the deliberation process by facilitating the participation of individuals from rural regions of both provinces. It also provided an equal opportunity for a diverse range of individuals to participate. Participants provided valuable information on the topics studied: the evaluation of the quality of the deliberation process and the postdeliberation self-perceptions of changes. Among other limitations, we observed that a few participants were passive in their participation; 5 (25%) out of 20 participants from Quebec and 9 (33%) out of 27 participants from Ontario did not share their opinion during the small group activity, while some of them did intervene during the plenaries and the training day by consulting the experts. During the training session, it was not possible to count each intervention from participants, as 1 small group session was not recorded. As a result, we only calculated the HHI for the deliberation session but not for the formative session. Some observer responses were more detailed than others’ responses, so the differences between their observations could not be compared.

Despite the possible biases associated with conducting an online deliberation process, we consider that it is an acceptable alternative when social distancing measures are necessary, such as during the COVID-19 pandemic. However, some unpredictable and organizational aspects must be taken into account to optimize the results. We believe that face-to-face deliberations will continue to be the first option to consider whether conditions are suitable for it.

### Conclusions

Overall, participants considered the quality of the ODD to be satisfactory. Among the quality dimensions evaluated, participants attributed the highest levels of satisfaction to learning new information, understanding information on issues of triage in extreme COVID-19 pandemic contexts, and the exercise of reflecting on the common good. We found a favorable evaluation of the ODD quality on the 4 main aspects included in the questionnaire: the process, the information, the reasoning, and the participation in videoconferencing. Some participants self-reported a change of opinion after the deliberation. The results of the questionnaire were consistent with the findings of the thematic analysis from the ODD transcripts. The online modality may be an acceptable alternative for DD in a pandemic context but with some organizational adaptations. More studies are needed to determine the feasibility and effectiveness of online deliberation processes.

## Appendix

Multimedia Appendix 1 Postdeliberation questionnaire.

Multimedia Appendix 2 Calculation of the Herfindahl-Hirschman Index.

Multimedia Appendix 3 NVivo coding query of participants who shared their perceptions about the quality of the online deliberation.

Multimedia Appendix 4 NVivo coding query of participants who wrote a comment on the self-perceived change of perspectives.

## Abbreviations

DD: democratic deliberation
HHI: Herfindahl-Hirschman Index
INM: Institut du Nouveau Monde
ODD: online democratic deliberation

## Data Availability

The datasets generated and analyzed during this study are available from the corresponding author on reasonable request.
