# Supplementary material for: Assessing the Quality of an Online Democratic Deliberation on COVID-19 Pandemic Triage Protocols for Access to Critical Care in an Extreme Pandemic Context: Mixed Methods Study
Source: J Particip Med. 2024 Nov 11;16:e54841. doi: 10.2196/54841 (PMC11589492; doi:10.2196/54841)
Supplement: Multimedia Appendix 1 [file jopm_v16i1e54841_app1.docx]

**Multimedia Appendix 1**. Postdeliberation questionnaire

**Section I**

Prioritization modalities and protocol content for adults and children were discussed in the deliberative process. Please answer the following questions based on your post-deliberation perspective.

**Assessment of post-deliberation perspectives**

**Question on change of perspective after deliberation on the protocols presented**

a) Do you consider that this democratic deliberation has led to a change in your perspectives? Please check the degree of change in your perspective, if applicable, regarding each of the following options.

| **Your perspectives on:** | **Totally** | **Partially** | **Somewhat** | **Not at all** |
| --- | --- | --- | --- | --- |
| 1) Clinical criteria of the adult protocol |  |  |  |  |
| 2) Principles and values of the adult protocol |  |  |  |  |
| 3) Pediatric/neonatal protocol principles and values |  |  |  |  |
| 4) The tie-breaker criteria |  |  |  |  |

a) Comments: ___________________________________________________________________________

**Section II**

**Tool for assessing the quality of the democratic deliberation** (Adapted from De Vries et al., 2011)

- Please rate the following items:

**Part I** (Process evaluation)

| **1. Facilitation**  How did you perceive your participation in the deliberation (sharing your perspectives in a video conference)? | **Very easy** | **Easy** | **Neutral** | **Difficult** | **Very difficult** |
| --- | --- | --- | --- | --- | --- |
| **2. Equal participation**  How did you perceive the opportunity to share your opinions and ask questions during the process? | **Very equal** | **Equal** | **Neutral** | **Unequal** | **Very unequal** |
| **3. Respect**  Did you feel respected in sharing your opinions with other participants in the process? | **Very respected** | **Respected** | **Neutral** | **Slightly respected** | **Not respected** |
| **3. Commitment**  How would you rate your engagement with the discussion group throughout the process? | **Very committed** | **Committed** | **Neutral** | **Slightly committed** | **Not at all committed** |

**Partie II** (Information evaluation)

| **1. Expert consultation**  How often do you estimate the number of times you consulted experts during the deliberation to obtain clarification? | **Very frequently** | **Frequently** | **Neutral** | **Infrequently** | **Not at all frequently** |
| --- | --- | --- | --- | --- | --- |
| **2**. **Use of incorrect information**  Do you consider that you received or shared incorrect information during the deliberation? | **Not at all** | **Not really** | **Neutral** | **A little** | **Totally** |
| **3. Learning new information**  How do you perceive your learning from the information obtained in the deliberation? | **Very satisfied** | **Satisfied** | **Neutral** | **Dissatisfied** | **Very dissatisfied** |
| **4. Understanding and applying the information**  How would you rate your understanding of the information presented in the deliberation? | **Very clear** | **Clear** | **Neutral** | **Unclear** | **Very unclear** |
| **5. Impact of information on opinions**  How do you perceive the impact of deliberative information on your opinions? | **Very influential** | **Influential** | **Neutral** | **Slightly influential** | **Not at all influential** |

**Part III** (Reasoning evaluation)

| **1. Rationale for opinion**  How would you rate your shared opinions in the deliberation? Were your opinions justified? | **Very justified** | **Justified** | **Neutral** | **Poorly justified** | **Not justified** |
| --- | --- | --- | --- | --- | --- |
| **2. Openness to complexity**  How would you rate your openness to the difficult topics discussed in the deliberation? | **Very open** | **Open** | **Neutral** | **Slightly open** | **Not at all open** |
| **3. Consideration of the societal perspective**  How did you perceive your consideration of the collective perspective (thinking for a common good) during the deliberation? | **Very considered** | **Considered** | **Neutral** | **Poorly considered** | **Not at all considered** |

**Part IV** (Videoconference evaluation)

| 1. How would you rate the audio quality or sound quality? | **Very satisfied** | **Satisfied** | **Neutral** | **Dissatisfied** | **Very dissatisfied** |
| --- | --- | --- | --- | --- | --- |
| 2. How do you rate the video quality (image quality)? | **Very satisfied** | **Satisfied** | **Neutral** | **Dissatisfied** | **Very dissatisfied** |
| 3. What is your assessment of the ease of use of video conferencing for deliberation? | **Very easy** | **Easy** | **Neutral** | **Difficult** | **Very difficult** |

a) Comments on the quality of online deliberation: ________________________________________________
