# Supplementary material for: Assessing the Quality of an Online Democratic Deliberation on COVID-19 Pandemic Triage Protocols for Access to Critical Care in an Extreme Pandemic Context: Mixed Methods Study
Source: J Particip Med. 2024 Nov 11;16:e54841. doi: 10.2196/54841 (PMC11589492; doi:10.2196/54841)
Supplement: Multimedia Appendix 2 [file jopm_v16i1e54841_app2.docx]

**Multimedia Appendix 2**. Calculation of the HHI

**Calculated HHI for Ontario group**

Calculation obtained from website: <https://goodcalculators.com/hhi-calculator/>


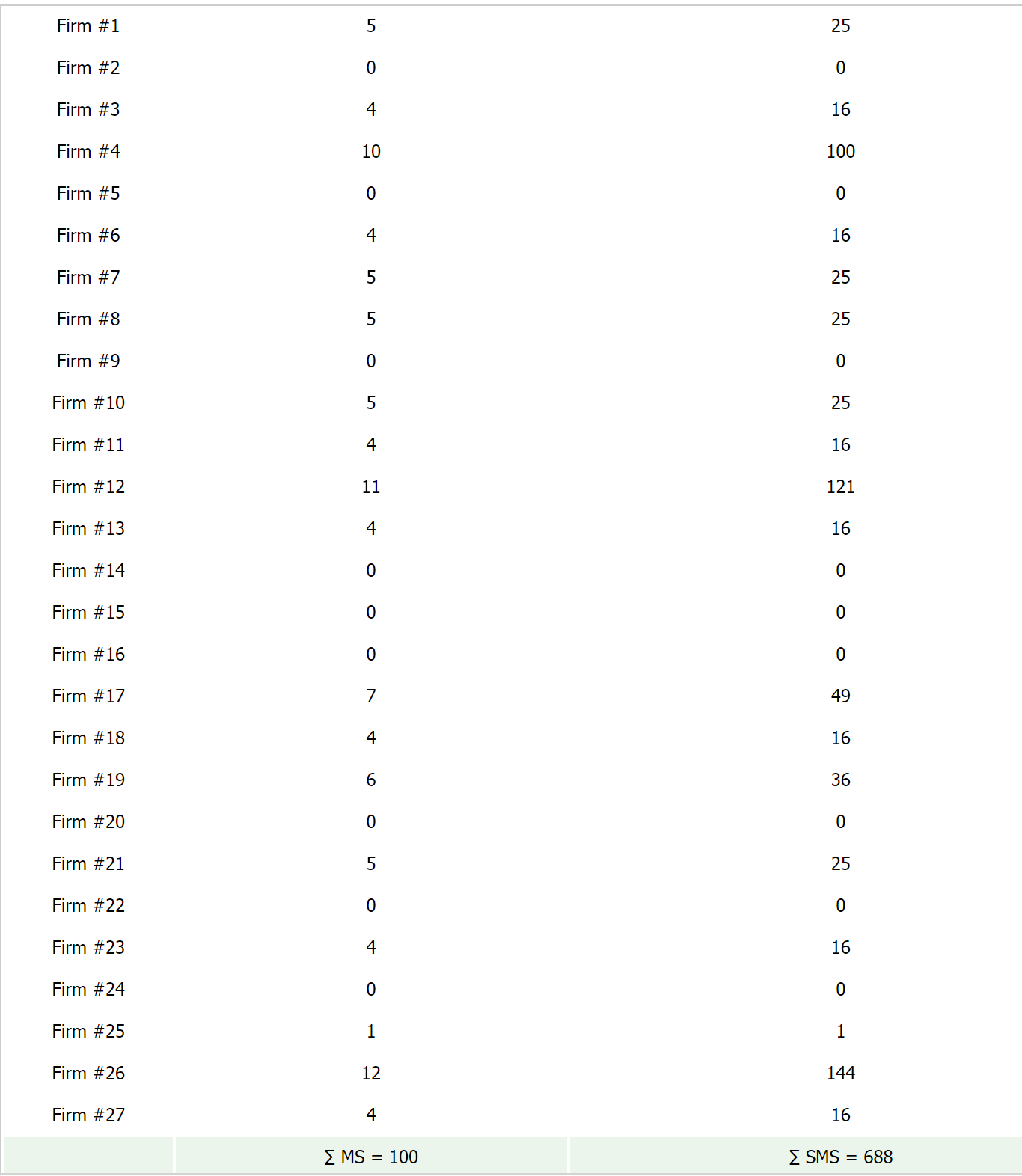


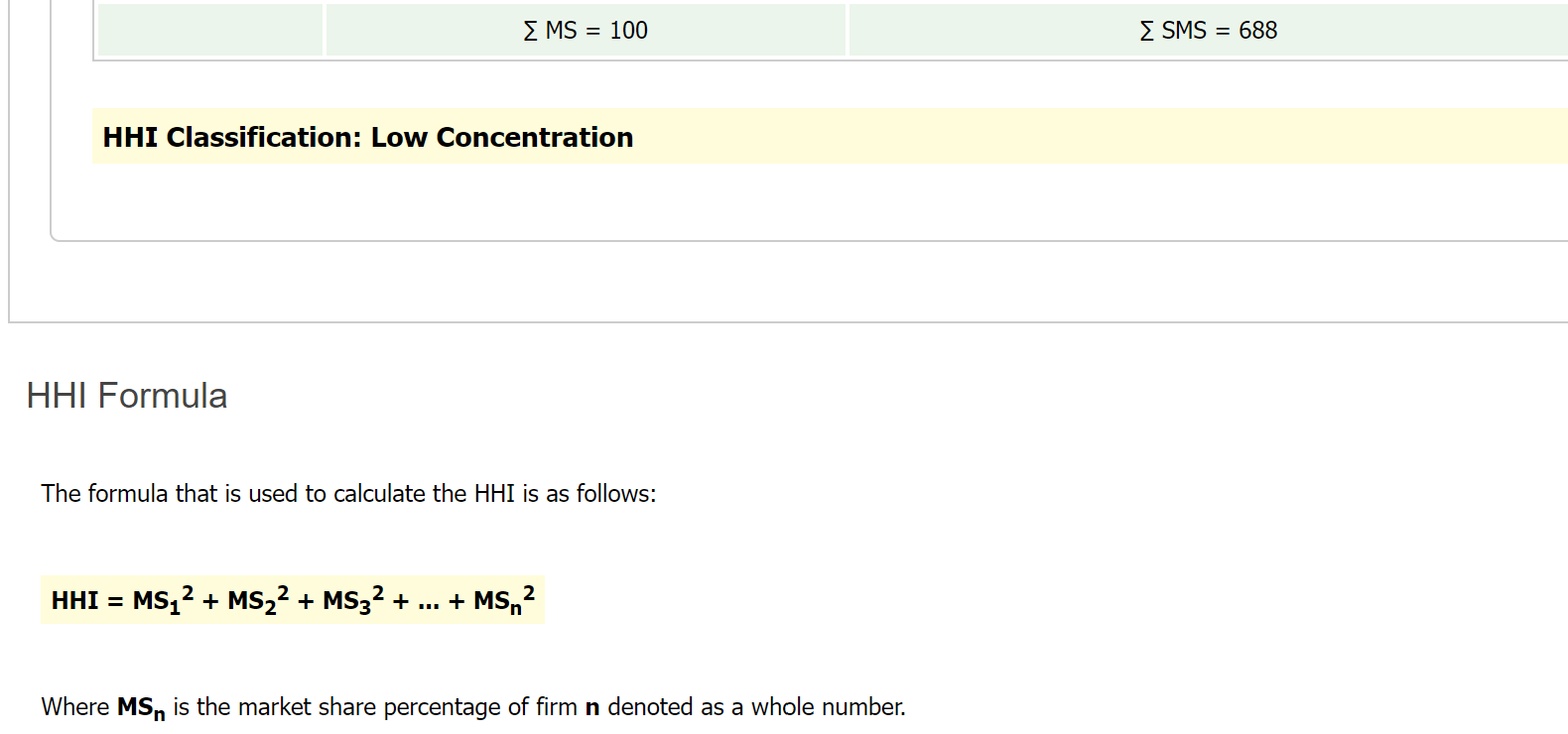


**Calculated HHI for Quebec group**


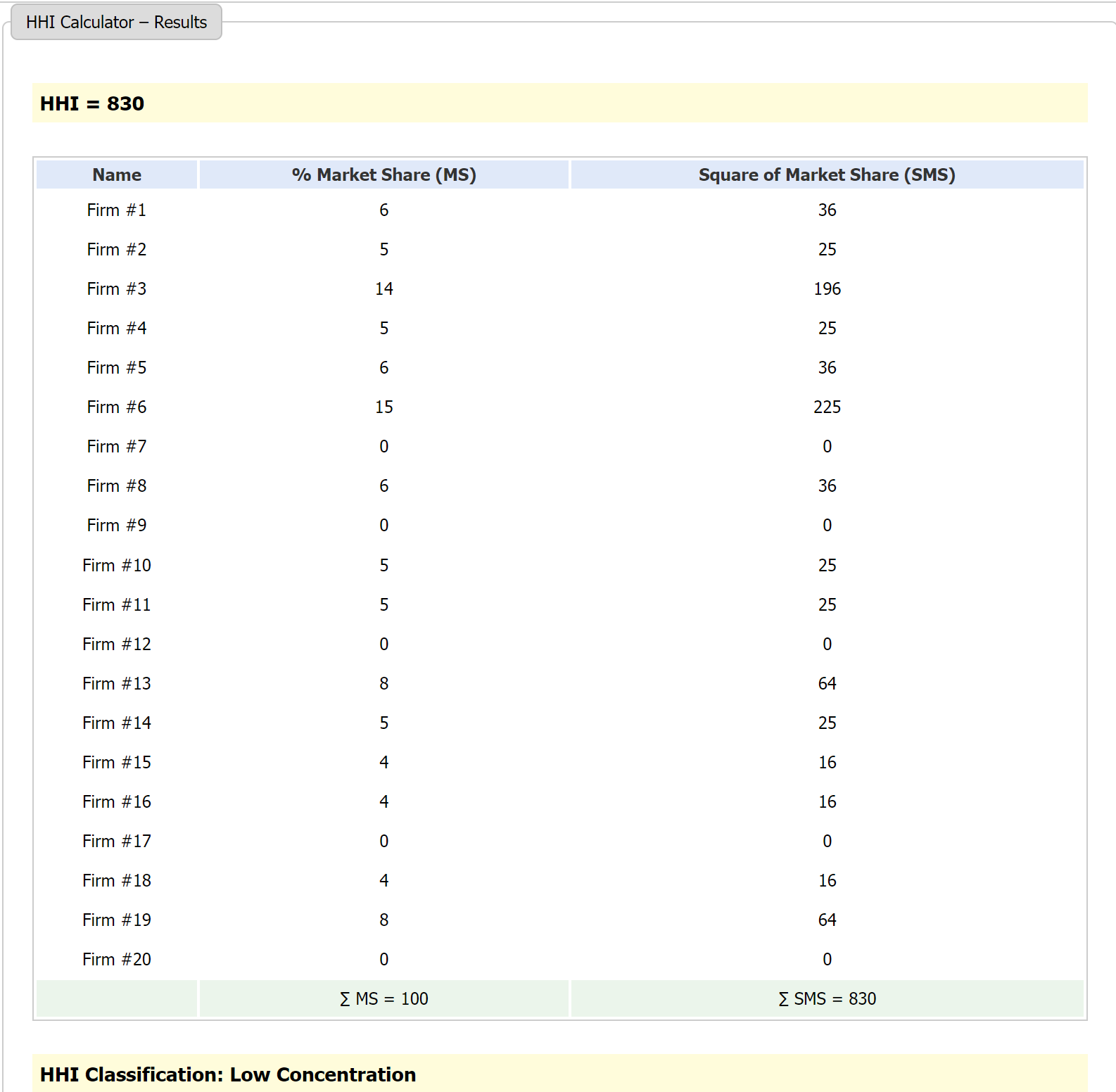


Regarding the formulas for calculating the HHI and the Normalized HHI:

***n***

**HHI = *∑ (X_i_ /X )^2^***

***i=1***

**Normalized HHI^N^ = HHI*−1/n***

***1−1∕n***

Where *X* represents the total number of spoken interventions from all participants in the deliberation; *Xi* represents the number of interventions by each participant; *Xi /X* is the proportion of interventions per participant, and *n* is the total number of participants in a deliberating group. A normalized HHI^N^ = 0 indicate a complete equality of spoken intervention, and an HHI^N^ = 1 a complete polarization of the deliberative dialogue [51, 52].

**Ontario and Quebec: Calculation of the Normalized HHI^N^**

**n= 27 (Ontario)**

**n= 20 (Quebec)**

**Ontario** HHI^N^= 0.069 – 0.037 = 0.032 = **0.033**

1 – 0.037 0.963

**Quebec** HHI^N^ = 0.083 – 0.05 = 0.033 = **0.035**

1 – 0.05 0.95
