# Supplementary material for: Assessing the Quality of an Online Democratic Deliberation on COVID-19 Pandemic Triage Protocols for Access to Critical Care in an Extreme Pandemic Context: Mixed Methods Study
Source: J Particip Med. 2024 Nov 11;16:e54841. doi: 10.2196/54841 (PMC11589492; doi:10.2196/54841)

**Multimedia Appendix 3**. NVivo coding query of participants who shared their perceptions about the quality of the online deliberation.


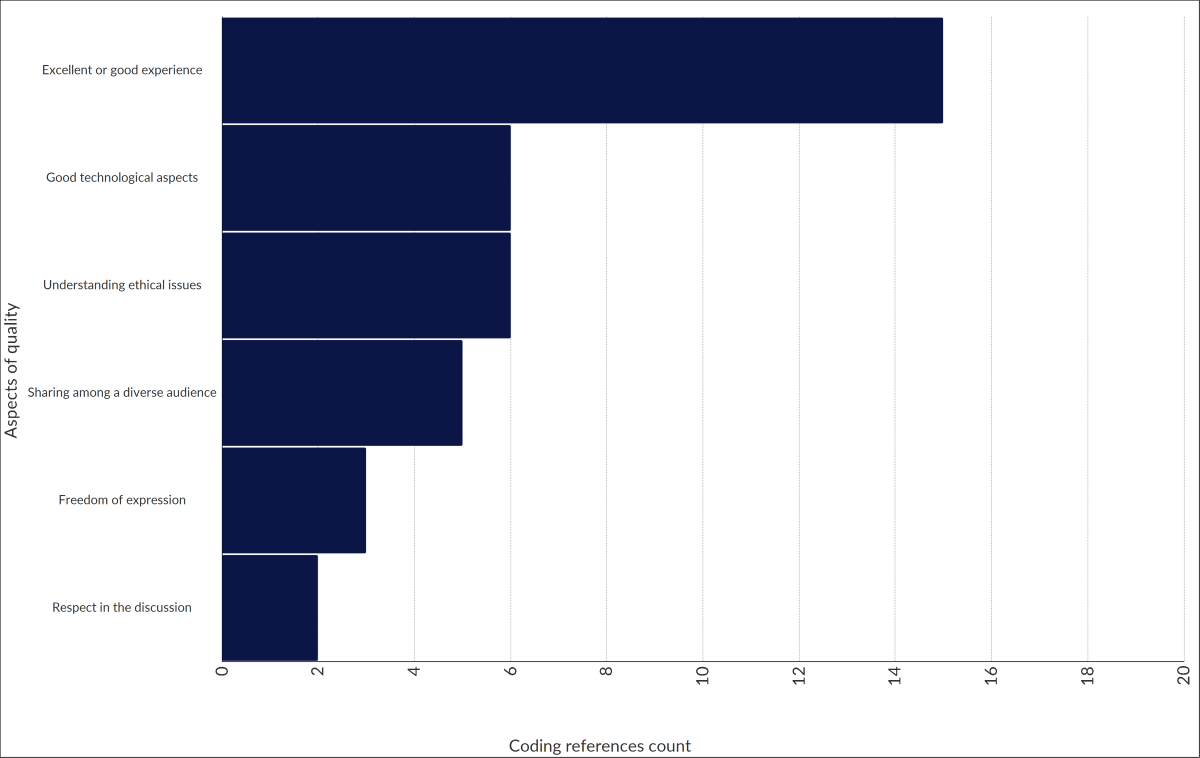

Supplement: Multimedia Appendix 3 [file jopm_v16i1e54841_app3.docx]
