# Supplementary material for: Assessing the Quality of an Online Democratic Deliberation on COVID-19 Pandemic Triage Protocols for Access to Critical Care in an Extreme Pandemic Context: Mixed Methods Study
Source: J Particip Med. 2024 Nov 11;16:e54841. doi: 10.2196/54841 (PMC11589492; doi:10.2196/54841)

**Multimedia Appendix 4**. NVivo coding query of participants who wrote a comment on the self-perceived change of perspectives.


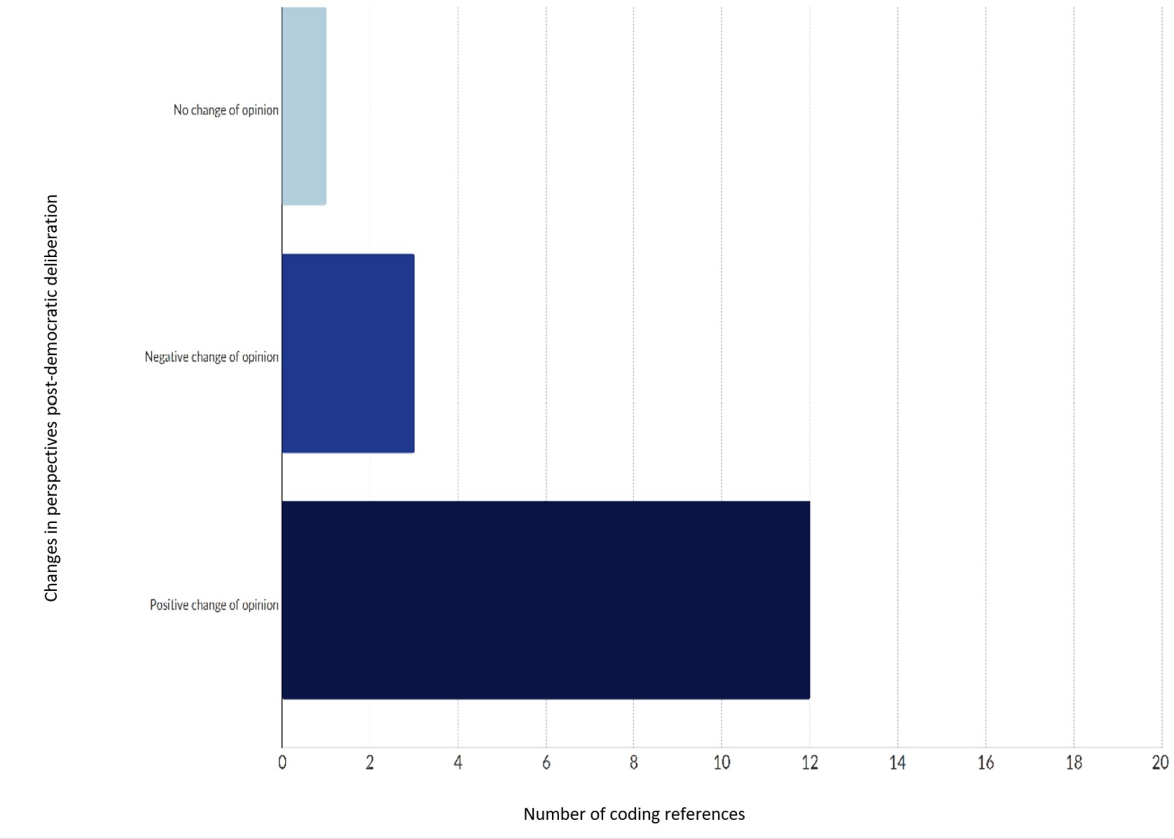

Supplement: Multimedia Appendix 4 [file jopm_v16i1e54841_app4.docx]
